# 3D electron tomographic and biochemical analysis of ER, Golgi and *trans* Golgi network membrane systems in stimulated Venus flytrap (*Dionaea muscipula*) glandular cells

**DOI:** 10.1186/s40709-018-0086-2

**Published:** 2018-08-08

**Authors:** Zachary R. Gergely, Dana E. Martinez, Bryon S. Donohoe, Soren Mogelsvang, Rachel Herder, L. Andrew Staehelin

**Affiliations:** 1 MCD Biology, University of Colorado at Boulder, Campus Box 347, Boulder, CO 80309 USA; 2 Instituto de Fisiología Vegetal (INFIVE), Universidad Nacional de La Plata–CONICET CC 327, La Plata, Argentina; 3 National Renewable Energy Laboratory, 15013 Denver West Parkway, Golden, CO 80401 USA; 4 Exxel Pharma, Inc, 12635 E Montview Blvd, Suite 100, Aurora, CO 80045 USA; 5 Wilson Sonsini Goodrich & Rosati, One Market Plaza, Spear Tower, Ste 3300, San Francisco, CA 94105 USA

**Keywords:** Venus flytrap, Golgi, *trans* Golgi network, Endoplasmic reticulum, Transmission electron microscopy, Electron tomography

## Abstract

**Background:**

The insect-trapping leaves of *Dionaea muscipula* provide a model for studying the secretory pathway of an inducible plant secretory system. The leaf glands were induced with bovine serum albumin to secrete proteases that were characterized via zymogram activity gels over a 6-day period. The accompanying morphological changes of the endoplasmic reticulum (ER) and Golgi were analyzed using 3D electron tomography of glands preserved by high-pressure freezing/freeze substitution methods.

**Results:**

Secretion of multiple cysteine and aspartic proteases occurred biphasically. The majority of the Golgi was organized in clusters consisting of 3–6 stacks surrounded by a cage-like system of ER cisternae. In these clusters, all Golgi stacks were oriented with their *cis*-most C1 cisterna facing an ER export site. The C1 Golgi cisternae varied in size and shape consistent with the hypothesis that they form de novo. Following induction, the number of ER-bound polysomes doubled, but no increase in COPII vesicles was observed. Golgi changes included a reduction in the number of cisternae per stack and a doubling of cisternal volume without increased surface area. Polysaccharide molecules that form the sticky slime cause swelling of the *trans* and *trans* Golgi network (TGN) cisternae. Peeling of the *trans*-most cisternae gives rise to free TGN cisternae. One day after gland stimulation, the free TGNs were frequently associated with loose groups of oriented actin-like filaments which were not seen in any other samples.

**Conclusions:**

These findings suggest that the secretory apparatus of resting gland cells is “overbuilt” to enable the cells to rapidly up-regulate lytic enzyme production and secretion in response to prey trapping.

**Electronic supplementary material:**

The online version of this article (10.1186/s40709-018-0086-2) contains supplementary material, which is available to authorized users.

## Background

The Venus flytrap, *Dionaea muscipula*, which evolved on nitrogen-poor soils, uses specialized snap trap leaves to capture insects and thereby obtain supplemental nitrogen for its growth [1]. Closure of the snapping leaves occurs in ~ 100 ms, and involves a snap-buckling instability mechanism [2]. Upon closure of the trap, secretory glands in the leaf surface are activated and induced to secrete digestive enzymes and highly hydrated sticky, mucilage-type enzyme-carrier molecules to break down the prey tissues into digestible compounds.

Darwin [3] performed experiments to determine which compounds were able to stimulate secretion by the glandular cells of *D. muscipula*. Based on his observations, he concluded that “…they do not secrete until excited by the absorption of nitrogenous matter” [3]. More recent studies have shown the sticky secretions to contain 1–2% protein [4], ~ 0.01% polysaccharides [5] as well as inorganic ions and water [1]. The lytic enzymes identified in the secretions include proteases, phosphatases, nucleases, chitinases and peroxidases [4, 6, 7]. More recently, a transcriptomic study of non-stimulated versus active traps identified, in addition to hydrolases, many stress pathway-associated proteins [8]. When trap closure and gland stimulation occur via the touch-sensitive hairs in the traps, the initial response involves the secretion of water, and hydrogen and chloride ions into the sealed traps [9]. This initial secretion reaches a steady state after 24 h, which coincides with the up-regulation of lytic enzyme secretion we report in this paper.

Although the Venus flytrap is one of only a small number of experimentally inducible secretory systems in plants [10, 11], remarkably little is known about the cell biology of the digestive gland cells in this plant, with most of the studies being carried out in the 1970s and 1980s [1]. At the ultrastructural level, thin section analysis of chemically fixed cells demonstrated the presence of all of the organelles known to be involved in the secretory pathway of plant cells, i.e. ER, Golgi, vacuoles and plasma membranes [4, 5, 7, 12, 13]. The anthocyanin pigment-containing vacuoles were also shown to accumulate proteins and lipidic compounds and to change their morphology upon induction [4, 12, 14]. In cells treated with [^14^C] leucine, autoradiography and biochemical experiments demonstrated the presence of newly synthesized proteins in the ER, Golgi, vacuoles and the cell wall [6, 14]. Based on these studies it was concluded that proteins synthesized in the endoplasmic reticulum (ER) were delivered both to the vacuoles and to the cell surface. However, due to the non-optimal structural preservation of the cells, the interpretation of the data was difficult, leading to unsubstantiated claims that secretion occurs via fusion of the ER with the plasma membrane or from vacuoles.

Recent progress in understanding the relationship between Golgi structure and function can be traced to two technological advances, cryofixation (high pressure freezing) and electron tomography [15]. High pressure freezing enables researchers to immobilize all cellular molecules within ~ 1 ms, which is fast enough to preserve most transient membrane events for ultrastructural analysis. Once frozen, the samples are freeze-substituted at − 80 °C prior to embedding in resin to preserve their 3D architecture [16]. Electron tomography, in turn, provides a means for increasing the z-axis resolution of the sample images from > 100 to 6–8 nm [17].

In most plant cells, the Golgi apparatus consists of dispersed, functionally independent Golgi stack/TGN (*trans* Golgi network) units encompassed by a ribosome-excluding matrix/scaffold [18]. The individual Golgi stacks are assembled from proteins and lipids produced in the ER and transported together with cargo molecules in COPII vesicles to the *cis*-side of the Golgi stacks [19, 20]. How the ER-Golgi interface is organized and how it functions are two topics that continue to attract a lot of interest and controversy [21, 22]. Each Golgi stack consists of 5–7 cisternae, which, in high pressure frozen/freeze-substituted cells, can be resolved into three types of cisternae—*cis*, medial and *trans*—based on their position in a stack, their geometry, and their staining characteristics [23]. These structural differences reflect differences in the functional activities of the different cisternal types [24–27]. Variations in Golgi architecture associated with the differentiation of root meristem cells into slime-secreting root cap cells have been correlated with differences in secretory pathway activities [28]. Transformation of the *trans*-most *trans*-Golgi cisternae into TGN cisternae also involves a complex set of maturational steps, including the recycling of ~ 35% of the membrane surface area, peeling of the cisterna from the stack, and packaging of the cargo molecules into secretory and clathrin-coated vesicles [29].

Most plant Golgi stacks travel along ER-anchored actin filaments with periods of rapid saltatory movements alternating with slower or wiggling motions [30–32]. During the rapid, linear translocation phases the Golgi move at speeds of up to 4 µm s^−1^, whereas during the wiggling or slower movement stages the translocation speeds are < 0.4 µm s^−1^. According to the “Dock, Pluck and Go” model of ER-to-Golgi trafficking [33], the slower movement stages correspond to the “Dock and Pluck” stages of the model and coincide with active ER export sites (ERES) capturing passing Golgi via COPII bud-associated scaffolding proteins that form links to the cis-side of the Golgi matrix, and then mediate the transfer of the COPII vesicles to the Golgi [23]. Golgi stacks coupled to an ERES via scaffolding proteins are often referred to as “secretory units” [21, 22, 34]. However, based on the fact that in Arabidopsis root tip cells the number of Golgi coupled to ERES is highly variable (15–70%) and not 100% [23], the secretory units must be viewed as transient and not permanent functional units.

All electron microscopy/electron tomography studies of high pressure frozen plant Golgi published to date have provided support for the cisternal maturation and not for the vesicle shuttle model of Golgi trafficking [22, 33, 35]. This model postulates that new cisternae are formed from COPII vesicles at the *cis*-most face of the Golgi stack, and then traverse the stack with their cargo as the mature TGN cisternae are shed from the *trans*-side of the stack [36]. New *cis* cisternae are generated from cisterna initials by the fusion of 3–5 COPII vesicles in contact with the C2 cisterna and grow by fusion with additional COPII vesicles [27]. COPI vesicles recycle membrane proteins in a retrograde direction to maintain the polar distribution of the cisternal enzymes across the stack. ER proteins are recycled exclusively from the *cis*-Golgi cisternae via COPIa-type vesicles, whereas COPIb-type vesicles are responsible for the recycling of membrane from Golgi-associated TGN to *trans*-, and from *trans*- to medial Golgi cisternae [27, 35, 37, 38]. Shedding of TGN cisternae has been documented via live cell imaging studies of tobacco BY-2 and Arabidopsis root and hypocotyl cells expressing fluorescently labeled marker proteins [39–41], and the associated structural differentiation of the TGN cisternae has been studied by means of electron tomography in cryofixed root tip cells of Arabidopsis [29].

The goal of this study was to reinvestigate the structural organization of the secretory pathway organelles in control and bovine serum albumin (BSA)-stimulated gland cells of the Venus flytrap using high pressure freezing and freeze-substitution techniques to preserve the cells, and electron tomography to derive information on the 3D architectural changes of ER, Golgi and TGN membrane systems. Our data demonstrate how the structural organization of these membrane systems changes during the BSA-induced synthesis and secretion of cysteine and aspartic acid proteases and of the sticky and highly hydrated slime molecules, the extracellular carriers of the lytic enzymes to the trapped prey and of the amino acids from the digested prey to the cells.

## Results

### Cryofixation of mature digestive glands of Venus flytraps is challenging

**Fig. 1:**
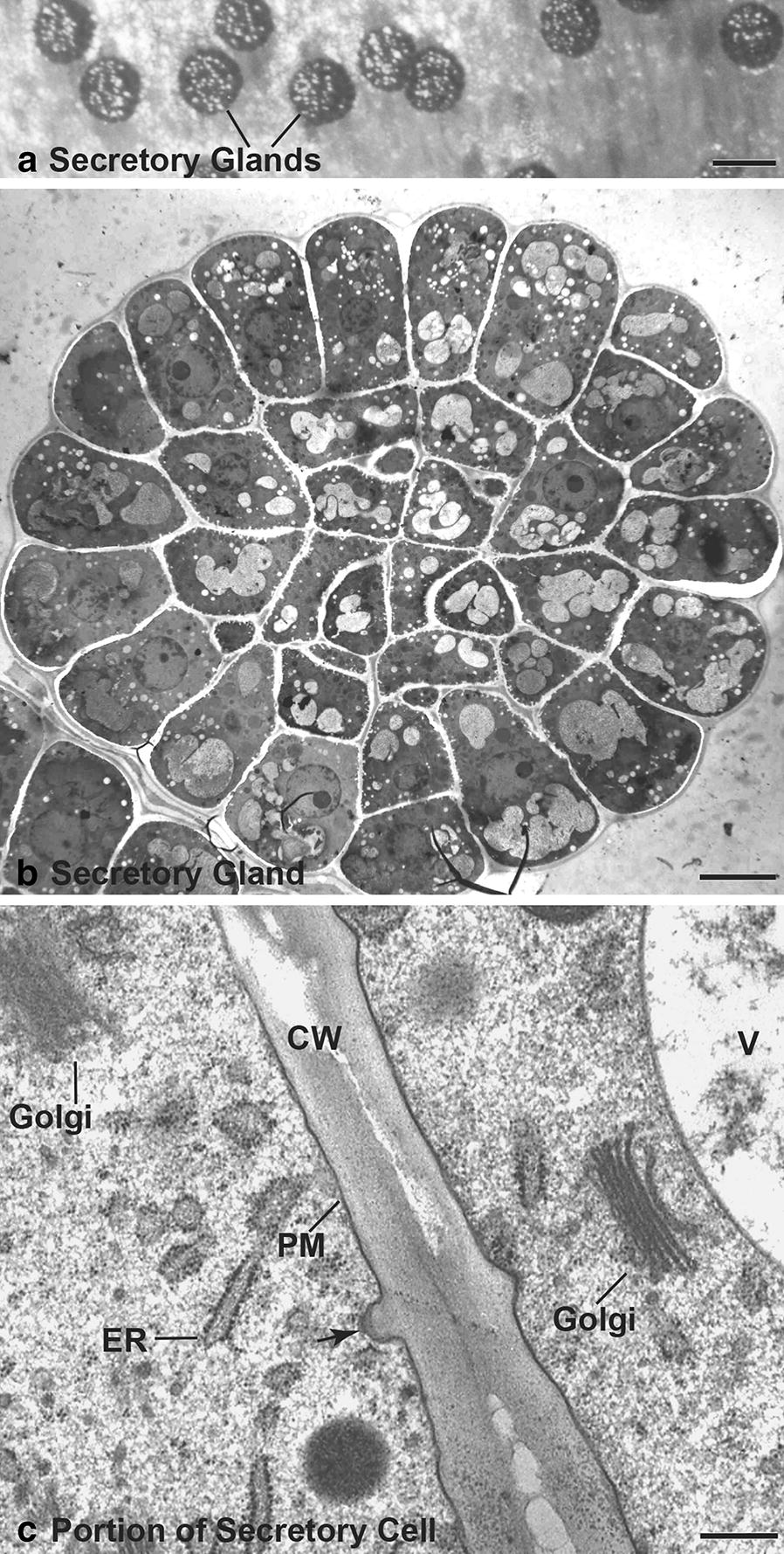
Venus flytrap glands and cells. **a** Light micrograph of the inner surface of a Venus flytrap snap lobe showing the stalked, digestive enzyme-secreting gland cells. Bar 100 µm. **b** Electron micrograph of a thin section through a digestive enzyme-producing gland preserved by high pressure freezing and freeze-substitution methods. The section plane is parallel to the lobe surface and shows the organization of the ~ 32 secretory cells within the gland. Bar 10 µm. **c** Higher magnification image of two secretory cells in a high pressure frozen/freeze substituted gland. Note the close apposition of the plasma membranes (PM) to the cell wall (CW) which is marked by cell surface area increasing protuberances (arrow). Endoplasmic reticulum (ER), vacuole (V). Bar 0.5 µm

Obtaining well-frozen samples of mature digestive gland cells of the Venus flytrap (Fig. 1a–c) is considerably more difficult than producing well-frozen cells of root tips and apical meristems [15, 29, 37], and required many more freezing runs to obtain well-preserved cells. This is probably due to the lower content of cytosolic solute molecules as evidenced by the low electron density of the stained cytoplasm and the low density of cytosolic ribosomes (Fig. 1c). The scarcity of ribosomes has somewhat limited our ability to morphologically define the boundaries of the Golgi/TGN encompassing scaffold systems [33], and in some samples the structural differences of COPIa- and COPIb-type vesicles were difficult to discern [27, 37].

### Stimulated digestive gland cells respond initially by secreting cysteine proteases then aspartic proteases

The digestive glands of the Venus flytrap consist of ~ 0.1 mm wide mounds of cells that protrude from the epidermis in the central region of the petiole lobes (Fig. 1a). They can be distinguished from the peripherally located alluring glands (nectaries) by the presence of red anthocyanin pigments in their vacuoles. Each gland consists of ~ 32 secretory cells (Fig. 1b). In non-stimulated glands, the cells contain numerous and often grouped Golgi stacks, rough ER cisternae, vacuoles, and cell walls with characteristic protrusions (Fig. 1c).

**Fig. 2:**
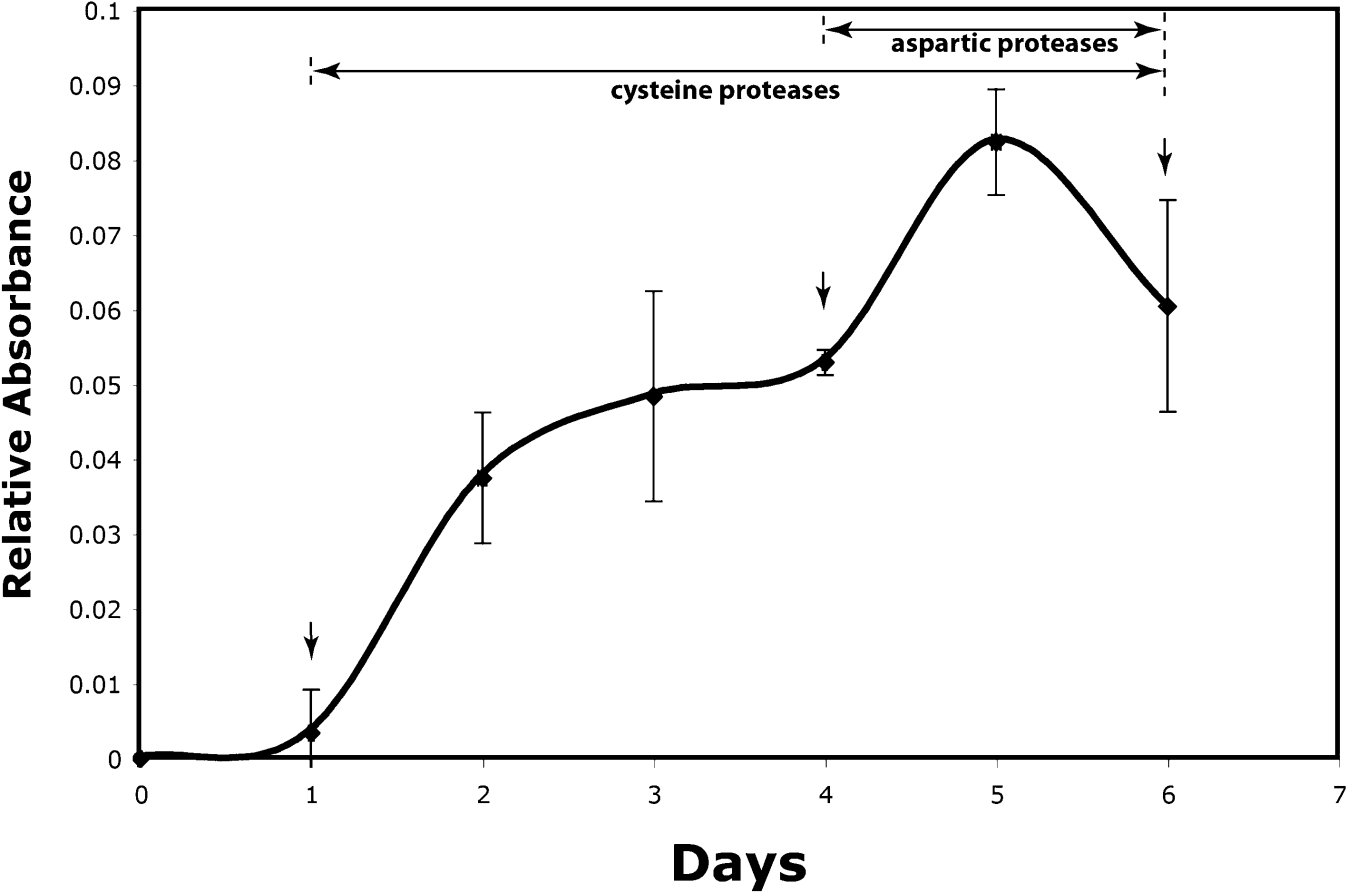
Changes in secreted protease activities after BSA stimulation of the secretory glands (fluorescent casein assay). Arrows indicate the time points where electron tomographic analysis was performed on stimulated samples. Note the biphasic nature of the secretory process. The type of proteases was determined using zymographs. Most traps reopened 7–10 days after induction

The secretory activity of the digestive gland cells was stimulated by the addition of 100 µL of 5% bovine serum albumin into each trap. Samples for the assays were collected at specific time points after addition of the BSA. The time course of protease secretion was determined by means of a fluorescent-casein-based general protease assay. The biphasic curve of secretion (Fig. 2) demonstrates that the secretory activity begins to ramp up on day 2 after stimulation, reaching a plateau between days 3 and 4, and then increasing again between days 4 and 5 before dropping off after day 5. Sticky slime became noticeable in the traps of the 3–6 day stimulated specimens.

**Fig. 3:**
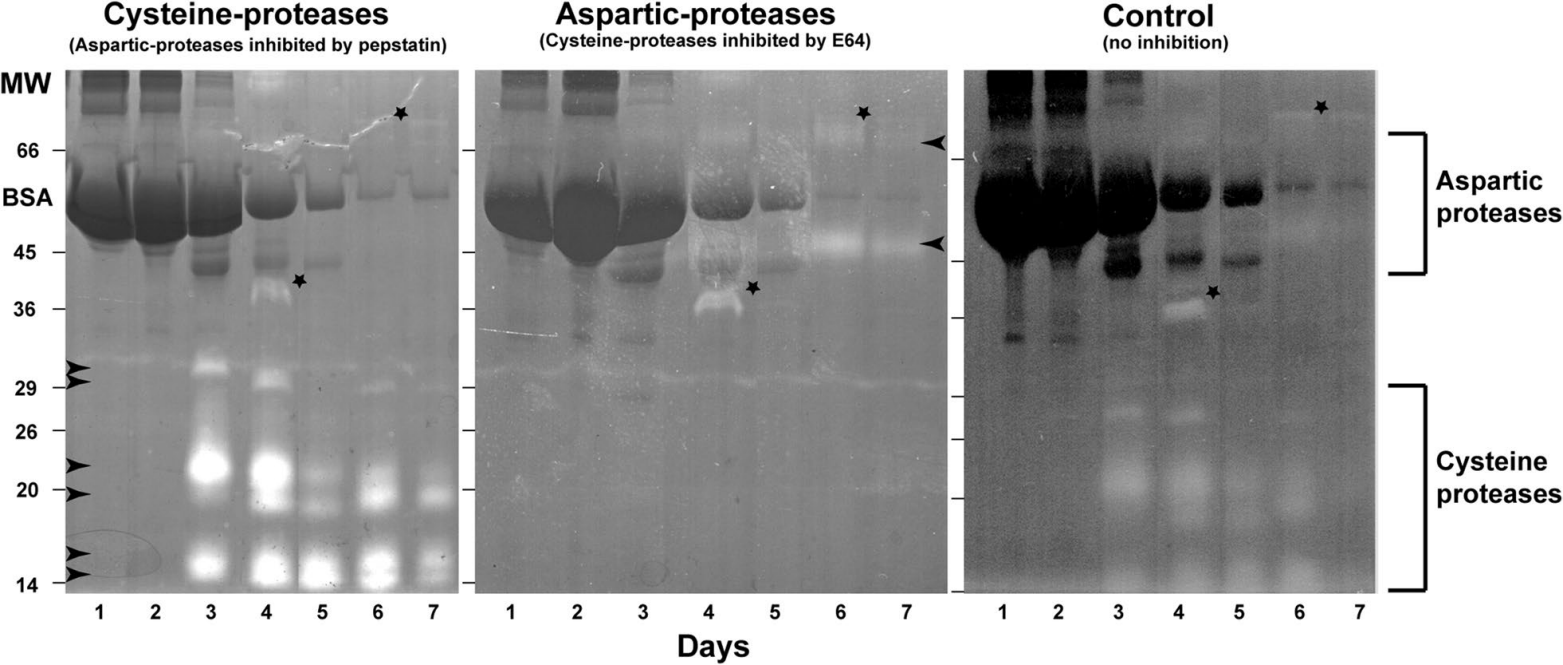
Zymogram gels of Venus flytrap eluates. The lighter stained gel bands mark sites of different proteases separated by electrophoresis. The distinction between cysteine and aspartic proteases is based on inclusion of two protease inhibitors, pepstatin and E64 in the gels. The control gel contained no inhibitors. The arrowheads mark the positions of reproducibly identified proteases. The starred bands are of unknown origin. Note the size differences of the cysteine and aspartic proteases

To characterize the types and numbers of different proteases secreted by the gland cells, we produced zymogram gels from days 1 through 7 (Fig. 3). The zymogram electrophoresis gels contained gelatin as a protease substrate within the acrylamide matrix. The gland samples were first solubilized and reversibly denatured in SDS, the proteins separated by gel electrophoresis, and the separated proteins renatured within the gels. Digestion clearing of the gelatin in the gels marks the positions of the individual proteases.

The zymograms show 6 or 7 bands on days 3–7, and possibly 2 faint bands on days 1 and 2 (Fig. 3). The course of degradation is also evidenced by the diminution of the ~ 60 kD BSA band over time. The activity of the four principal proteases that appear on day 3, reaches a peak on day 4 and then gradually declines. These proteases have apparent molecular weights of ~ 14, ~ 16, ~ 20 and ~ 23 kD. Two other protease bands at ~ 32 and ~ 30 kD are seen at days 3 and 4, respectively. Since these proteases are inhibited by the specific protein inhibitor E64, they are identified as cysteine proteases. In contrast to the lower MW cysteine proteases, the higher MW aspartic proteases at ~ 50 and ~ 90 kD only appear to be secreted in enhanced quantities during days 6 and 7 (Fig. 3).

### A majority of the gland cell Golgi are organized in small clusters with their *cis* cisternae associated with an ER export site on the surrounding ER

**Fig. 4:**
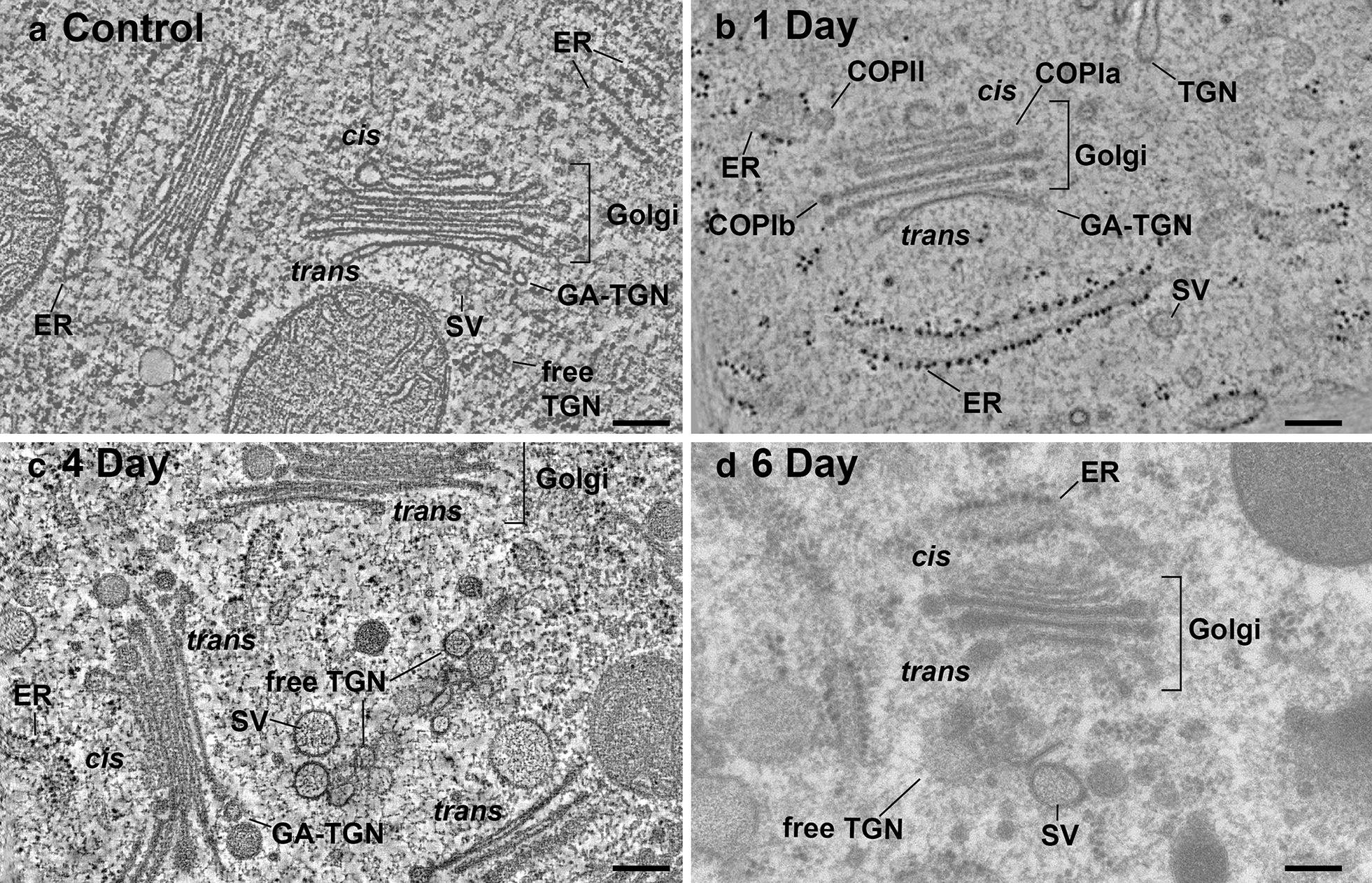
Electron micrographs of Golgi stacks in high pressure frozen/freeze-substituted Venus flytrap gland cells. Note the increase in size of the inflated cisternal margins of the Golgi-associated *trans* Golgi network (GA-TGN) and the free TGN elements and of the size of the secretory vesicles (SV) in the 4 and 6 day samples compared to the control and 1 day samples. Peeling GA-TGN cisternae are seen in **a**–**c**, and free TGN cisternae in **a**, **c** and **d**. In **b**, a COPII bud is seen at an ER export site adjacent to the *cis*-side of the Golgi stack, and COPIa- and COPIb-type vesicles are associated with *cis* and medial Golgi cisternae. Bars 0.2 µm

**Fig. 5:**
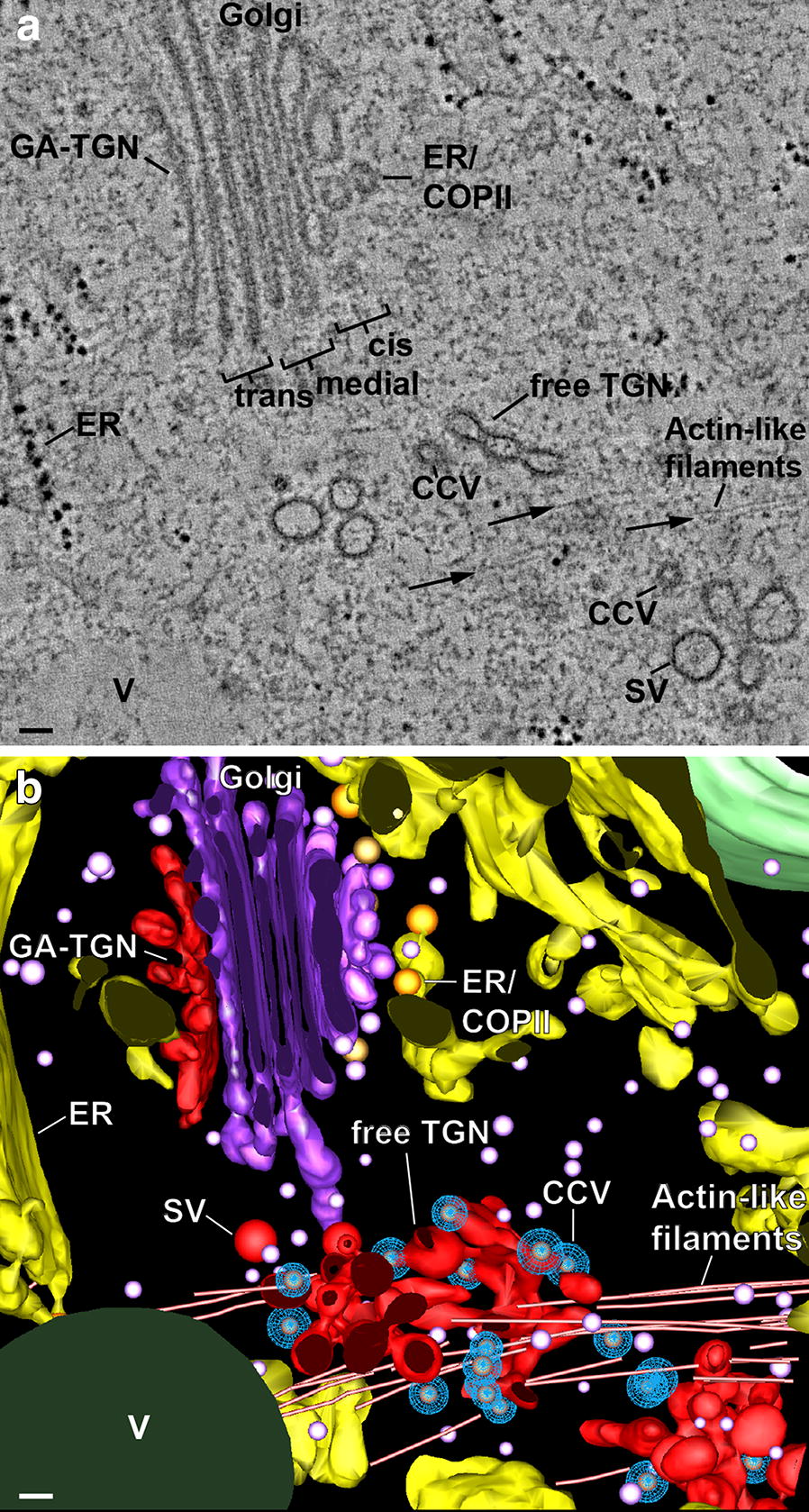
Tomographic slice image and corresponding tomographic model of a Golgi stack and TGN cisternae with associated actin-like filaments (arrows) in a 1 day-induced gland cell. **a** Tomographic slice image illustrating differences in architecture and staining of *cis,* medial and *trans* Golgi, Golgi-associated *trans* Golgi network (GA-TGN) and free trans Golgi network TGN cisternae, an ER cisterna with a budding COPII vesicle (ER/COPII), clathrin-coated vesicles (CCV), and secretory vesicles (SV). The actin-like filaments associated with the free TGN cisternae are a unique feature of the 1 day BSA-induced cells. **b** 3D tomographic model of the structures illustrated in **a**. The filaments are organized in the form of a loose bundle around the free TGN cisternae (only one 1 day-induced sample was analyzed in this study). Vacuole (V). Bars 0.1 µm

**Fig. 6:**
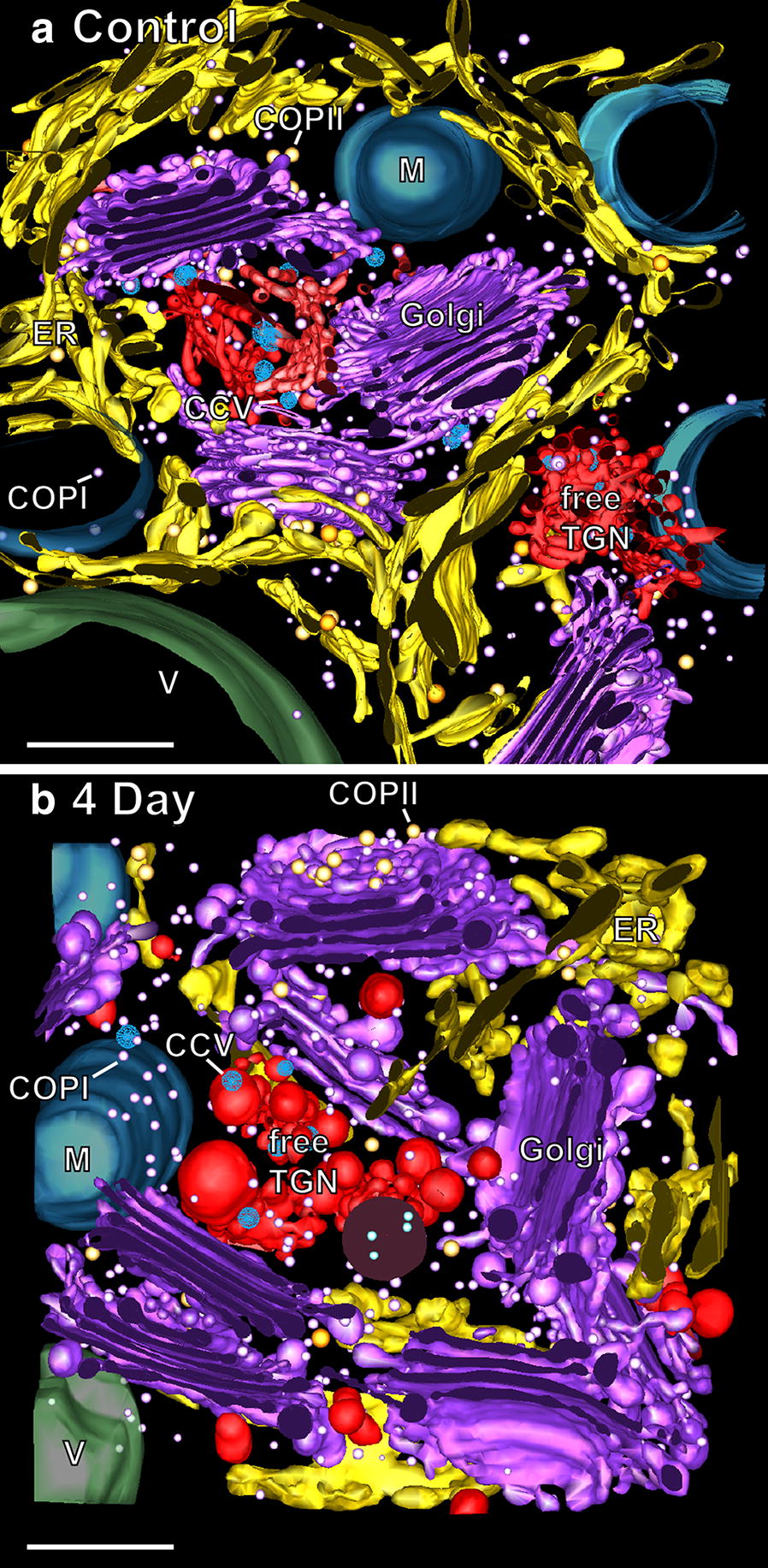
Tomographic reconstructions of clustered Golgi stacks and associated ER cisternae in a control and a 4 day-induced Venus flytrap gland cell. **a**, **b** Tomographic models of clustered Golgi stacks in a control cell (**a**) and a 4 day-induced gland cell (**b**) derived from a serial tomograms (1 µm × 2 µm × 2 µm volumes). The purple Golgi stacks appear clustered with their *cis*-sides facing ER export sites in the encompassing ER membranes (yellow). The red colored free TGN cisternae are somewhat displaced from the *trans*-sides of the stacks. COPII-type vesicles (yellow), COPI-type vesicles (white), clathrin-coated vesicles (CCV, blue), mitochondrion (M, blue), vacuole (V, green). Bars 0.5 µm

**Fig. 7:**
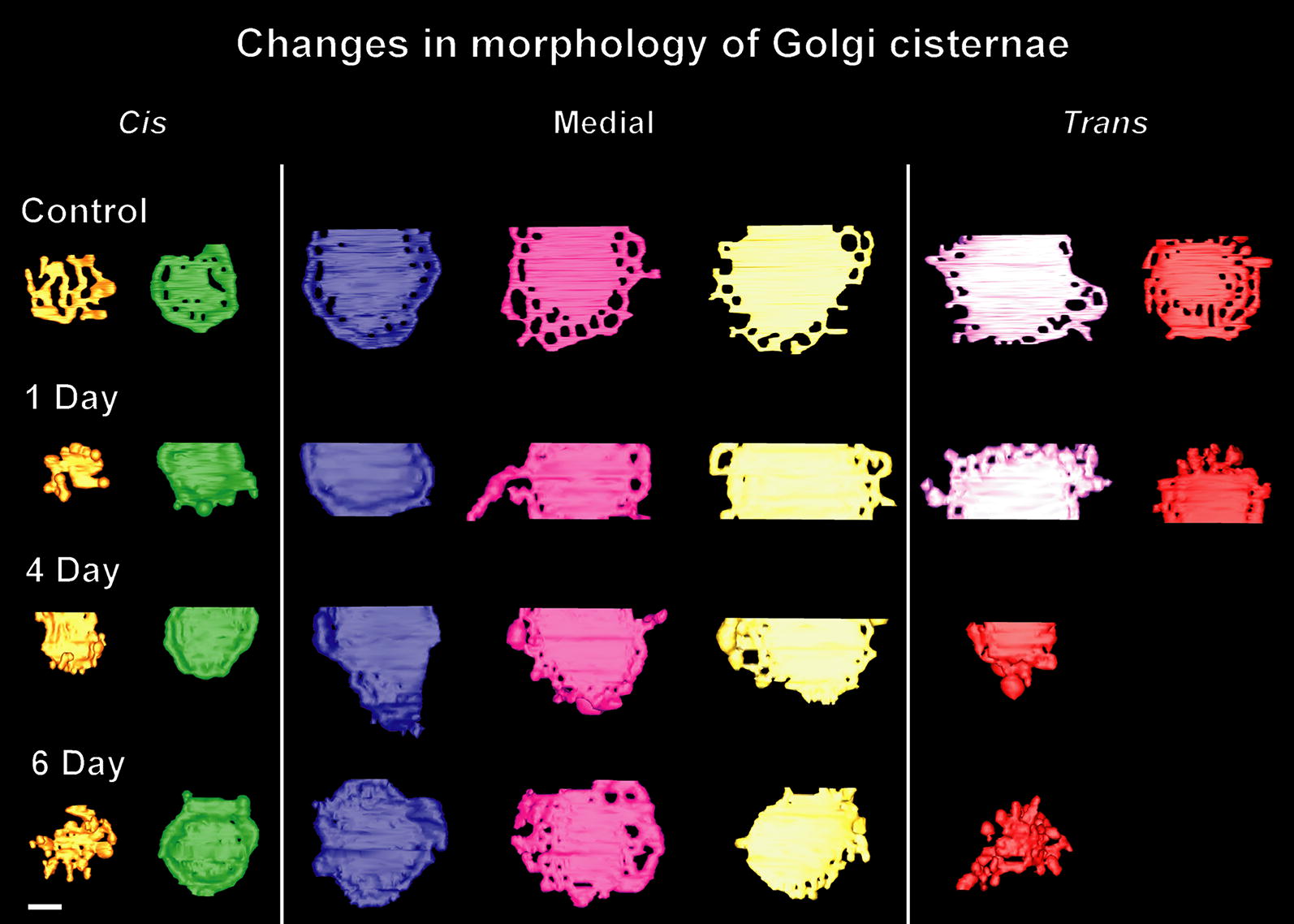
Gallery of tomographic reconstructions of Golgi stack cisternae of control and stimulated Venus flytrap gland cells. The individual cisternae (C1–C7) are viewed from the *cis*-side of the stacks. The C1 *cis*-cisternae exhibit the greatest variability in size and architecture. Control (non-stimulated) Golgi cisternae typically possess many peripheral fenestrae, whereas the BSA-stimulated Golgi possess few. BSA stimulation also caused a reduction in the number of cisternae (days 4, 6) and an increase in size of the bulbous cisternal margins that give rise to budding secretory-type TGN vesicles. Bar 0.2 µm

The ER membranes and Golgi stacks of the glandular cells are readily seen in electron micrographs of cryo-fixed/freeze-substituted cells due to the light staining of the cytosol (Figs. 1c, 4, 5a). A majority of the Golgi stacks are organized in small clusters (3–6 stacks) in both non-stimulated and BSA-stimulated cells (Fig. 6). No actin-like filaments or cables were associated with the Golgi clusters. The individual Golgi stacks exhibit a typical plant Golgi morphology (Figs. 4, 5) with each stack displaying a *cis*-to-*trans* cisternal polarity due to differences in cisternal morphology and staining. These differences are seen most clearly in the tomographic slice image Fig. 5a and in the corresponding tomographic model (Fig. 5b), which also illustrates the 3D organization of the associated ER and TGN cisternae. Variations in the 3D architecture of the individual Golgi cisternae of control and BSA-stimulated cells is documented in the face-on model views of Fig. 7 and Additional file 1: Figure S1.

**Fig. 8:**
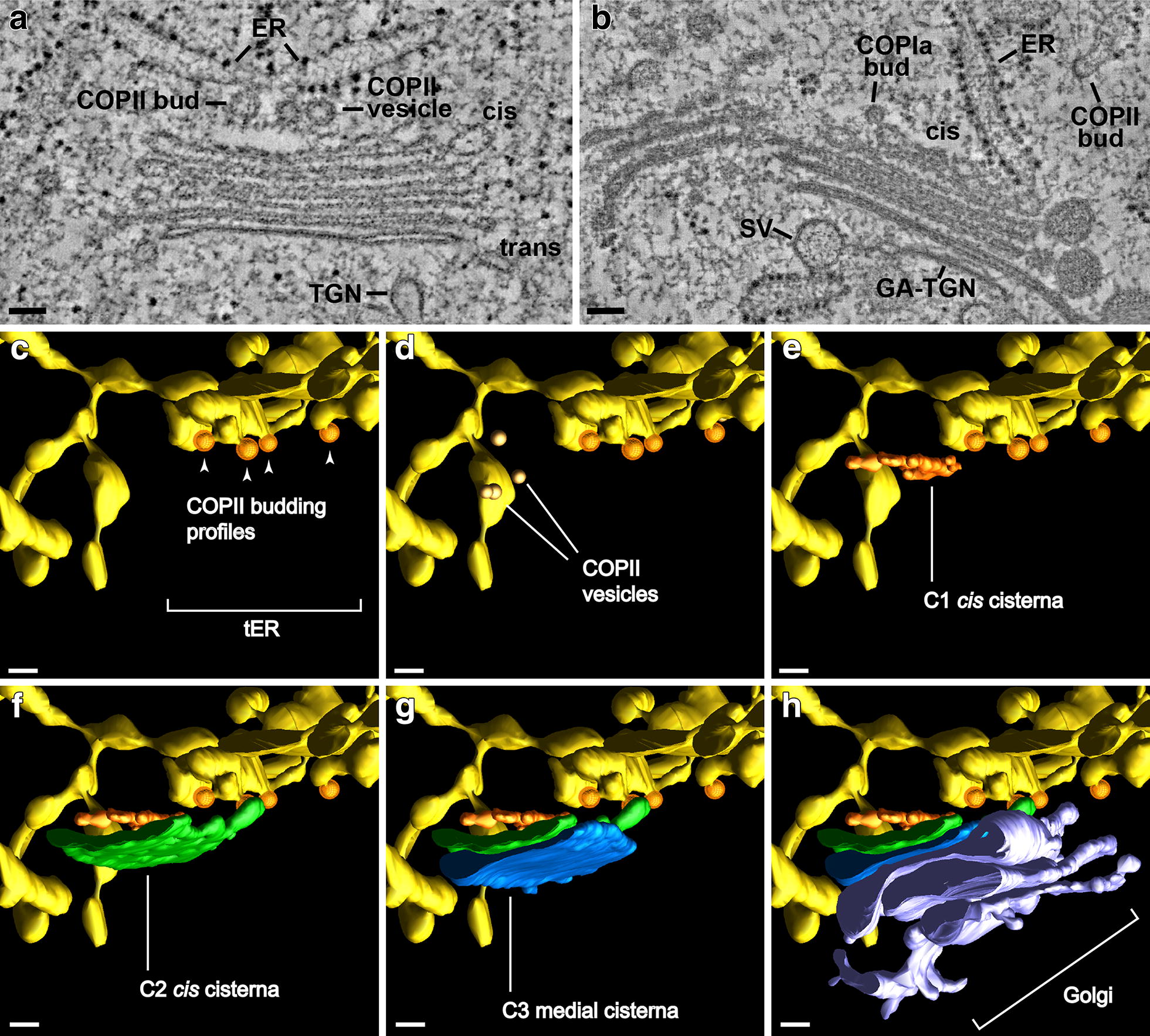
ER (yellow) export sites with COPII buds and vesicles and associated *cis*- and medial Golgi cisternae. **a** Tomographic slice image illustrating a budding and a detached ~ 70 nm in diameter COPII vesicle sandwiched between an ER export site and the *cis*-side of a Golgi stack. **b** Tomographic slice image of an ER export site with a COPII vesicle bud, and a *cis*-Golgi cisterna displaying a ~ 45 nm in diameter COPIa vesicle bud. **c**–**h** Tomographic reconstructions illustrating the 3D architecture of an ER export site with budding and free COPII-type vesicles together with the C1 (orange), C2 (green), C3 (blue) cisternae, and remaining cisternae (purple) of a Golgi stack. Note that the ER region shown possesses two COPII vesicle-producing sites separated by a short tubular ER domain with the one on the right (**c**) producing COPII buds and the one on the left (**d**) exhibiting 4 COPII vesicles sandwiched between the ER cisternae and adjacent C1 and C2 *cis* cisternae (**e**, **h**). Bars 0.1 µm

The Golgi clusters are surrounded by ribosome studded, rough ER cisternae organized in the form of cage-like structures that extend tubular extensions into the spaces between the stacks (Fig. 6). Most striking, all of the Golgi stacks are oriented with their *cis*-cisternae facing an ERES defined by the presence of ~ 60 nm in diameter budding and free COPII vesicles sandwiched in a ~ 250 nm wide zone between the two membrane systems. On average, each ERES in non-stimulated cells possessed ~ 2 COPII-type budding structures and ~ 7 COPII-type vesicles. The ERES depicted in Fig. 8c–e illustrates two other interesting features: the site appears to be composed of two adjacent but independently operating COPII vesicle-producing sites separated by a tubular cisterna, with the site on the right consisting of 4 budding COPII vesicles, and the one on the left having no COPII buds but a cluster of 4 free COPII vesicles (Fig. 8d), all of which are confined to the narrow space between the two membrane systems.

### Gland cell Golgi stacks possess a typical plant cell Golgi architecture, but the variability in staining patterns often blurs the differences between cisternal types

**Fig. 9:**
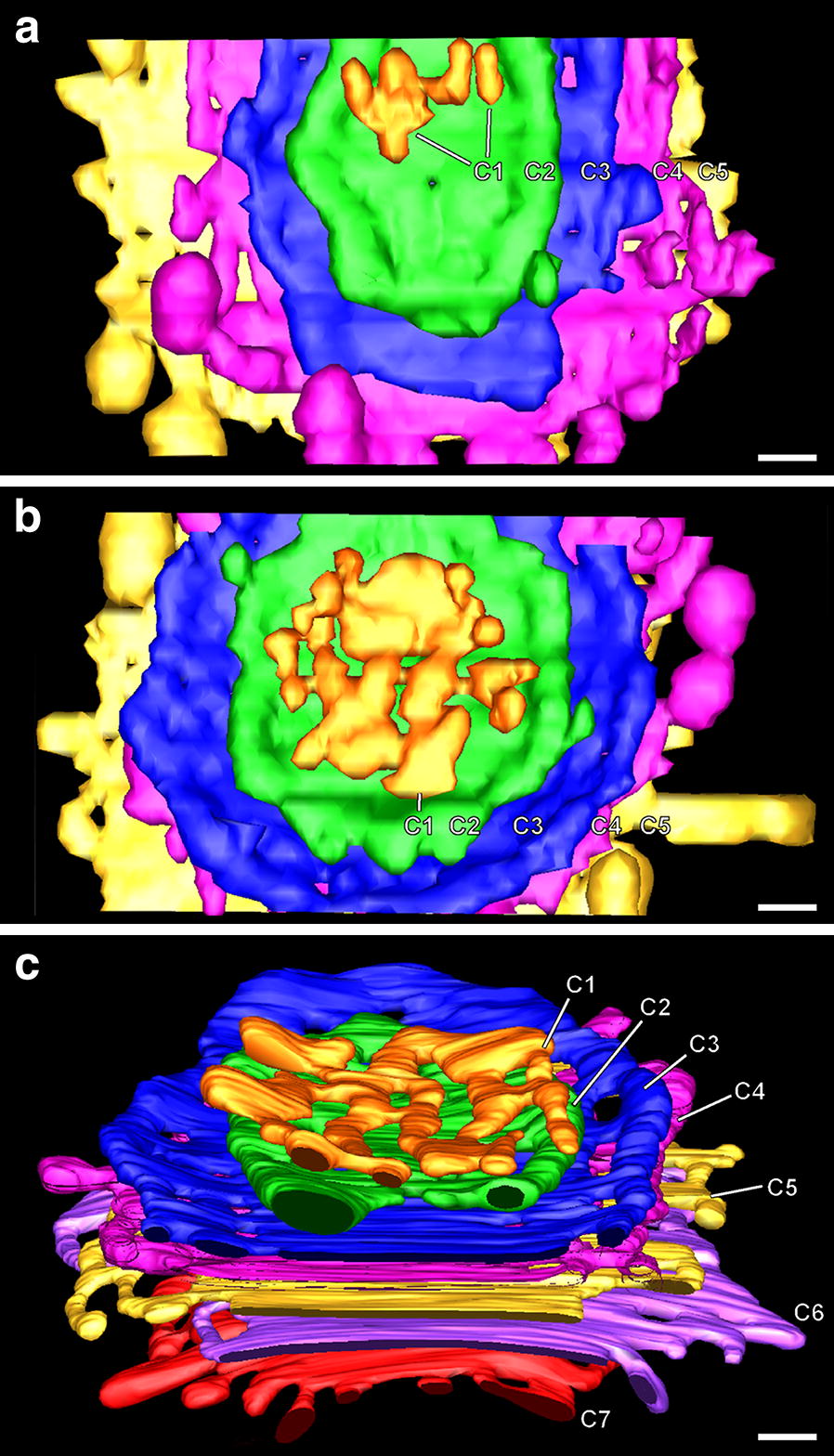
Tomographic models illustrating the variability in size and geometry of C1 *cis*-Golgi cisterna initials. The cisternae are color coded: C1 (orange), C2 (green), C3 (blue), C4 (pink), C5 (yellow), C6 (purple), C7 (red). The smallest C1 cisterna initial seen in **a** has a surface area equivalent to 3–4 COPII vesicles. The larger cisterna initials (**a**–**c**) possess branched, tubular architectures with many tubules exhibiting COPII vesicle-size spherical ends. The Golgi stacks depicted in **a** and **b** are shown in face-on views, and in **c** the stack is presented at an angle to highlight both the geometry of the C1 cisterna and the spatial organization of the stack. Bars 0.1 µm

*Cis*-Golgi cisternae can be identified based on multiple structural criteria [23, 27, 28], including their position in the stack, their lightly stained lumen, their highly variable size and geometry, and their greater thickness. Based on these criteria, each Venus flytrap gland cell Golgi possesses two *cis* cisternae, with the *cis*-most (C1) cisternae exhibiting the greatest amount of variability in both size and morphology (Figs. 4, 5, 7, 9). The smallest C1 *cis*-cisterna initials have a surface area equivalent to ~ 3 COPII vesicles, the intermediate size ones exhibit a characteristic lobed-type of architecture, while the largest ones assume an increasingly coherent, disc-shaped morphology (Fig. 9). All of the C2 *cis* cisternae possessed a disc-like geometry and were intermediate in size between the C1 *cis* and the following C3–C5/C6 medial and *trans*-Golgi cisternae (Fig. 7). Due to the paucity of cytosolic ribosomes, the outer boundary of the Golgi scaffold [23] is difficult to discern, but the absence of ribosomes within a 40–60 nm wide layer around the Golgi stacks (Figs. 4b, c, 5a) is consistent with the presence of such a scaffolding system. In our 19 tomograms we have found no evidence for tubular membrane connections between ER and Golgi cisternae as reported for chemically fixed and zinc iodide/osmium tetroxide stained plant cells [22].

The C2–C5 (C6–C7) *cis*, medial and *trans* gland cell cisternae in all but the 6-day BSA-induced cells possess a remarkably flat, parallel geometry, with all of the swollen cisternal domains confined to the disc margins (Figs. 4, 5, 6, 7, 8, 9). Distinguishing medial- and *trans*-Golgi cisternae in thin sections of both control and BSA-stimulated cells is somewhat challenging since the structural features defined in other cell types [27, 28] are not always as clearly developed in the mature gland cells, and the ratios of the different cisternal types seems to vary. For example, the characteristic collapsed luminal geometry of *trans*-Golgi cisternae is sometimes seen only in the *trans*-most Golgi and the following Golgi-associated TGN cisterna (Fig. 6a, b).

**Fig. 10:**
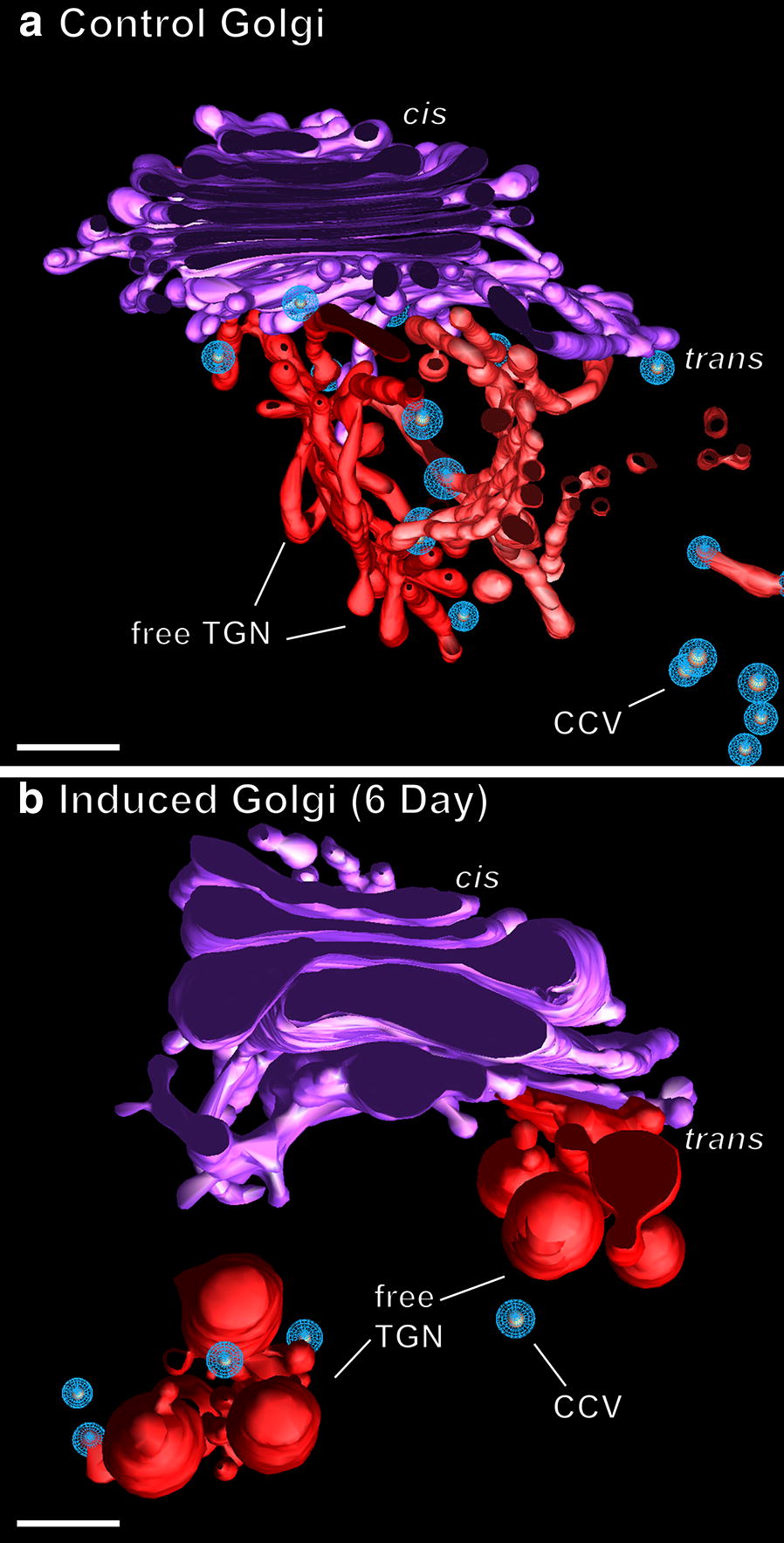
Comparison of Golgi stacks and TGN cisternae of a control (**a**) and a 6 day-induced (**b**) Venus flytrap gland cell. Note the general swelling of the Golgi cisternae (purple) of the induced stack compared to the control stack. Similarly, the budding vesicles of the induced TGN cisternae (red) are much larger than those produced by the more slender, tubular TGN networks of the control Golgi. The number of clathrin-coated buds and vesicles (CCV) formed by the induced TGN cisternae is considerably reduced compared to the control TGN. Bars 0.2 µm

In contrast, the transition between the *trans*-most Golgi cisterna and the adjacent Golgi-associated TGN cisterna can be readily defined using the structural criteria of Kang et al. [29]. Most notably, the Golgi-associated TGN cisternae exhibit a distinct cisternal peeling geometry and a reduction in size (Figs. 4a, b, 5). Free TGN cisternae are usually seen in the general vicinity of the *trans*-side of the Golgi stacks and give rise to clathrin-coated vesicles (Figs. 5, 10). In Golgi clusters, the specific Golgi stack that gave rise to a given free TGN cisterna is not always discernable (Fig. 6).

### BSA-induced activation of gland cells causes a doubling of ER-bound polysomes and swelling of ER and Golgi cisternae, but does not increase the number of budding or free COPII vesicles

In Figs. 4a–d, 5, 8 and 9 we document the changes in ER, Golgi and TGN architecture that occur in response to stimulation of the digestive enzyme-secreting glands with BSA. Structurally, the increase in biosynthesis of the lytic enzymes manifests itself in a doubling in density of ER-bound ribosomes from ~ 5 to ~ 10 ribosomes per 300 nm of ER tomographic slice image transect (Figs. 4, 6). In contrast, no change in the spatial relationship between ERES and *cis*-Golgi cisternae was seen, and no increase in the number of COPII vesicle-budding profiles or ~ 60 nm in diameter COPII vesicles at ER export sites was observed (Figs. 6, 8). Similarly, the striking increases in Golgi and TGN cisternal volumes (Fig. 10) were not matched by significant changes in surface area of the individual medial- and *trans*-Golgi cisternae (~ 550,000 nm^2^ per cisterna for control and ~ 590,000 nm^2^ per cisterna for the 4 and 6 day induced cells).

Swelling of the cisternal rims of Golgi stacks in slime-secreting root cap [28], border [42], and seed coat [43] cells is caused by the accumulation of mucilage cargo molecules that give rise to the sticky slime when secreted/rehydrated. BSA-induced cisternal swelling is most pronounced in the margins of the disc-shaped, medial and *trans* cisternae (Figs. 4c, d, 6, 10), and the luminal staining pattern is identical to the staining pattern seen in mucilage secreting root cap, border and Arabidopsis seed coat cells. Face-on views of the individual, modeled cisternae highlight additional changes in cisternal architecture (Fig. 7; Additional file 1: Figure S1). Most notably, the cisternae of the control Golgi stacks exhibit a characteristic ring of medium size fenestrae just internal to the swollen margins. These fenestrae are first seen in the C2 *cis* cisternae, become most pronounced in the medial cisternae, and disappear in the *trans*-Golgi cisternae. Within 1 day of BSA stimulation, most of the fenestrae in the C2 *cis* and the medial cisternae disappear, while swelling of the margins of the medial and *trans* cisternae increases. By 4 days, the cargo molecules begin to preferentially accumulate in the marginal domains, giving rise to large, elongated cisternal bulges. Simultaneously, the number of Golgi cisternae per stack decreases from 6 to 5, and the morphological differences between medial and *trans*-Golgi cisternae become blurred. These pronounced changes continue in the 6-day samples, where the number of Golgi cisternae is often reduced by one (Figs. 4d, 10; Additional file 1 Figure S1).

### BSA treatment induces changes in TGN architecture, but only in the day 1 induced samples are the free TGN cisternae associated with oriented actin-like filaments

As illustrated in the tomographic models (Figs. 5, 8, 9), BSA treatment caused major changes in TGN morphology. Thus, while the free TGN cisternae in the control cells exhibit a branched tubular architecture, whose origin can be traced to the cisternal fenestrae (Fig. 7), those of the induced cells are comprised of more cisterna-like compartments, whose morphology is dominated by large, forming secretory vesicles (Figs. 6b, 10b). These bulbous TGN compartments often seem to break up into two or three pieces before the individual large secretory vesicles are released. BSA induction also leads to a major reduction in the number of clathrin-coated buds and vesicles produced by free TGN cisternae (for comparison, see Fig. 10a, b).

As shown in a previous study [29], mature TGN cisternae that have separated from the originating Golgi stack become independently mobile before they fragment and release their vesicles. The same type of displacement of free TGN cisternae away from the Golgi stacks is also evident in Venus flytrap gland cells (Figs. 4d, 10). Unexpectedly, in the 1-day samples—but not detectable in any of the control, or the 2–6 day cells analyzed by electron tomography—the free TGN cisternae are surrounded by loose bundles of parallel actin-like filaments that cross the cytoplasm (Fig. 5). Since these filament bundles extended beyond the reconstructed volumes of our tomograms, we were not able to determine the end points and the 3D organization of the filaments in the cytoplasm and thereby obtain information about the possible role of these filaments in TGN cisterna and vesicle trafficking.

## Discussion

### Cysteine proteases predominate during the first half of the digestive period and aspartic proteases are secreted near the end

The Venus flytrap gland cells used in our experiments took 6 days or longer to digest the 5% BSA we supplied. Differentiation of the Golgi stacks in the induced tissues started on day 1 and the induced morphology was maintained through day 6 (Figs. 4b–d, 6, 7). This contrasts with the timing reported by several other groups. For example, in a study of chemically fixed cells stimulated with 6% bactopeptone, the induced Golgi changes were seen only for 1–2 days [7]. More recently, triggering of the snap trap closure response by living prey has been demonstrated to be mediated by mechano-electric hair cells that generate action potentials [44]. Furthermore, flytrap plant cells can count the number of action potentials produced by a trapped insect with > 5 needed to immediately activate the protein synthesis and secretion pathways in the closed traps [45, 46]. After 24 h, flytrap leaves stimulated in this manner were found to have up-regulated 3447 genes and down-regulated 2826 genes [8].

The results from the zymogram gels (Fig. 3) are consistent with the results of the time course of the protease activity assays (Fig. 2) in that they demonstrate that not only the quantity, but also the types of proteases secreted vary during the course of flytrap digestion. The zymograms resolve 6–7 protease bands versus 4 in previous biochemical assays of flytrap eluant [6]. The differences could be related to the fact that we assayed extracts from 6 consecutive days after stimulation, rather than the initial extract only. Alternatively, our gels could have better resolution. Finally, our flytraps were stimulated with BSA instead of other nitrogenous compounds used in earlier studies. Flytraps may be able to secrete different sets of proteases in response to different stimuli.

As seen from the intensity of the bands in the control gel (Fig. 3), cysteine proteases appear to be the most abundant proteases secreted, followed by aspartic proteases. Coincidently, of the more than 600 different proteases identified in the *Arabidopsis thaliana* genome, cysteine proteases are more prevalent than aspartic proteases [47]. Aspartic proteases have been identified and cloned in the digestive fluids of many carnivorous plants including *Nepenthes* and *Drosera* species [48, 49]. However, this is the first report of aspartic proteases in *Dionaea*. Similarly, cysteine proteases have not been reported previously in the Venus flytrap. Since we were unable to detect any inhibition of proteases by EDTA or phenanthroline in our gels, we have been unable to confirm the presence of Carboxypeptidase A, a metallo protease in our zymogram time-series. For this reason we have been unable to confirm the claim [6] that metallo-enzymes are an important component of the peptide hydrolase activity in *Dionaea* secretions. Instead, we postulate that a battery of cysteine and aspartic proteases are the major enzymes responsible for the peptide hydrolase activity in *Dionaea*.

### BSA-induced activation of flytrap gland cells produces changes in ER cisternae but no changes in ER export sites

As reported previously, induction of the *D. muscipula* gland cells causes de novo protein synthesis, an increase in protein secretion, and an increase in secreted hydrolytic enzymes, particularly proteases [4, 13]. The protease activities data presented in Figs. 2 and 3 are consistent with these earlier results and support the notion that the BSA-induced stimulation of the gland cells causes an increase in the biosynthetic activities of the membrane systems of the secretory pathway. Structurally, this increase in biosynthetic activities manifests itself in several ways, a doubling in density of bound ribosomes from ~ 5 to ~ 10 ribosomes per 300 nm of ER membrane transect in tomographic image slices, and swelling of Golgi and TGN cisternae (Figs. 4, 6, 7, 10). Equally interesting and somewhat puzzling, however, are the findings that neither the number of COPII-type vesicle budding profiles per ERES, nor the number of COPII-type vesicles increased in parallel with the increased rate of secretion. What this suggests is that rather than expanding the surface area of the ER to produce more secretory proteins, the cells make better use of the existing ER membranes by increasing the number of bound polysomes. Similarly, the absence of a BSA-induced increase in COPII buds/vesicles indicates that in non-stimulated cells the COPII vesicle transport system between the ER and the Golgi has spare capacity. Alternatively, the velocity of the COPII-mediated transport activities could be up-regulated. The first hypothesis is consistent with the observation that in tobacco leaf epidermal cells induced to produce more soluble proteins for export the number of ERES did not increase, whereas an increase in ERES numbers occurred in cells stimulated to produce more integral membrane proteins [50]. The observation that deflagellation-induced up-regulation of scale synthesis and secretion in the alga *Scherffelia dubia* also does not lead to an increase in COPII-type buds and vesicles [27], suggests that our Venus flytrap results are not unique.

There are conflicting reports on the ratio of ER export sites (ERES, COPII budding sites) to Golgi stacks and their spatial relationship to each other in plant cells [51]. According to one study [52] in which the ERES were visualized by means of fluorescently labeled SAR1p and Sec12p expressed in live tobacco leaf cells, most of the marker molecules moved together with mobile punctate bodies (Golgi bodies). This led to the suggestion that Golgi stacks are permanently coupled to ERES and function as “secretory units”. However, when the ERES of tobacco BY-2 cells were labeled with SEC13:GFP, the number of ERES greatly exceeded the number of Golgi bodies [53]. Furthermore, the moving Golgi temporarily associated with the ERES, suggesting that the Golgi stacks were not continuously linked to ERES. These differences have now been explained by a study of Arabidopsis root tip cells involving EM tomography [23], which demonstrated that in meristem cells 70% of the Golgi stacks were coupled to ERES, and in columella cells only 15%. Thus, both the ratio of ERES to Golgi stacks and the percentage of Golgi stacks coupled to ERES varies in a cell type specific manner. Taken together, these findings support the “Dock, Pluck and Go” model of Golgi trafficking [33]. Although all of the COPII-budding profiles associated with the clustered Golgi stacks analyzed in this study were located within 250 nm of a C1 *cis* cisterna and thereby would classify as secretory units, the fact that we did not systematically investigate the spatial relationship between COPII-budding sites and dispersed Golgi stacks precludes us from drawing more general conclusions about the percentage of Golgi stacks coupled to ERES in the gland cells of the Venus flytrap.

### The structural assembly intermediates of *cis*–Golgi cisternae and of forming TGN cisternae support the cisternal maturation model of Golgi trafficking

The mechanism of intra-Golgi trafficking has remained a controversial topic of Golgi research for the past 30 years. The two principal models are the vesicular shuttling and the cisternal maturation model [35, 36, 38]. Both of these models make specific structural predictions pertaining to the membrane activities at the *cis*- and *trans*-faces of the Golgi stacks. Thus, the vesicle shuttle model postulates that both the *cis*-most and the *trans*-most cisternae are conserved entities that are stably associated with the stacks, whereas the cisternal maturation model predicts the presence of cisternal assembly intermediates at the *cis*-face and of shedding cisternae at the *trans*-face of the stacks.

The electron tomography-based reconstructions of the *cis*-Golgi cisternae illustrated in Figs. 7, 9 and Additional file 1: Figure S1 provide clear evidence for differences in size and shape of the *cis*-most (C1) cisternae that support the structural predictions of the cisternal maturation model. In particular, the models show that the smallest cisterna initials that assemble on the surface of the C2 *cis*-cisternae are equivalent in size to ~ 3 COPII-type vesicles (Fig. 9a; Additional file 1: Figure S1), that the intermediate size, branched, tubular C1 cisternae form by the fusion of initials with each other and with additional COPII vesicles (Fig. 9b, c), and that the largest ones (Fig. 7, day 4 model) appear disc-like and approach the size of a C2 cisterna. All of these structural features together with the absence of luminal staining in the *cis*-cisternae are consistent with the hypothesis that *cis* cisternae are assembly structures that do not participate in biosynthetic functions [27].

Similarly, our *trans* Golgi and TGN images and models frequently depict cisternal elements in the process of peeling from the stacks and becoming free TGN cisternae (Figs. 4, 6, 10) as suggested by live cell imaging [39–41] and electron tomography studies [29]. Separation of the *trans*-most cisternae from the stacks appears to coincide with the transformation of the disc-shaped cisternae (Fig. 7) into 3D tubular networks (Figs. 4a, b, 5b, 10a) or 3D clusters of product-filled swollen vesicles connected through constricted tubular membrane domains (Figs. 5b, 10b; [33]). Interestingly, unlike in Arabidopsis root tip cells [29] where clathrin-coated buds were seen both on Golgi-associated and free TGN cisternae, we only observed clathrin-coated vesicles forming on free TGN cisternae in the flytrap gland cells. In NRK (normal rat kidney) mammalian tissue culture cells, clathrin-coated buds also appear to be confined to a single (*trans*-most Golgi/TGN) cisterna [54]. Together, these data provide direct support for the cisternal maturation model of Golgi trafficking.

### The swelling of the BSA-induced flytrap gland Golgi and TGN cisternae is caused by up-regulation of slime and not protease synthesis

The Golgi of slime-secreting plant cells were among the first to be studied in plants and these experimental systems provided a straightforward means for correlating the dynamics of slime secretion and Golgi architecture [55]. The first such correlative studies focused on the carnivorous plant *Drosophyllum*. This plant captures insects by a sticky slime that is produced by the Golgi in the stalked glands and digested by lytic enzymes secreted into the slime droplets by the surrounding sessile gland cells [56–58]. Numerous other researchers have noted the presence of sticky slime in the digestive enzyme fluids secreted by several other types of carnivorous plants, including the closed traps of stimulated Venus flytrap plants reviewed in Juniper et al. [1].

The BSA-induced changes in Golgi architecture in the Venus flytrap gland cells resemble to a significant extent those associated with the differentiation of root tip meristematic cells to columella cells, and to early and late types of slime-secreting root cap and border cells of tobacco, alfalfa and Arabidopsis [28, 42], as well as the structural changes accompanying the development of the slime-producing Golgi in Arabidopsis seed coat cells [43]. These morphological changes include the development of inflated cisternal margins and the swelling of entire Golgi cisternae due to the accumulation of fine-filamentous, osmiophilic mucilage molecules, the blurring of structural differences between medial and *trans* Golgi cisternae, and the reduction of the number of cisternae per stack (Figs. 4c, d, 6b, 10b). Unexpectedly, and unlike in root cap and border cells, the development of the slime-producing Golgi stacks in the gland cells does not include the formation of intercisternal elements [28, 42]. In contrast to the cisternal swelling associated with the stimulation of the BSA-induced production of lytic enzymes and slime molecules in flytrap cells, the gibberellic acid (GA3) mediated induction of α-amylase, protease and ribonuclease synthesis and secretion in barley aleurone cells [59], which occurs without a concomitant activation of slime synthesis, produced enlarged ER cisternae but no swelling of Golgi or TGN cisternae [60]. Together, these results support the hypothesis that the pronounced swelling of the BSA-induced Golgi and TGN cisternae in *Drosophyllum* gland cells is caused by the synthesis of the mucilage molecules and not by the synthesis of the lytic enzymes.

### Actin-like filaments associated with free TGN cisternae were only observed in the 1 day post BSA induction gland cells

The presence of loose arrays of actin-like filaments associated with the free TGN cisternae exclusively in the 1-day samples (Fig. 5) was one of the least anticipated findings of our study. This is also the first report describing Golgi/TGN-associated actin-like filaments in electron tomographic micrographs. Noteworthy is the fact that the filaments are relatively short, do not contact each other laterally, but are still aligned. In addition, the filaments do not seem to be directly attached to the TGN membranes but associated with ribosome-excluding cytoplasmic material surrounding the cisternae (TGN matrix/scaffold proteins) [33]. In mammalian cells, actin filaments not organized into bundles are short-lived and ~ 200 nm long [61].

The functional significance of the discovery of TGN-associated actin-like filaments exclusively in the 1 day samples remains a mystery, but the following observations suggest that this association might speed up delivery of the first enzymes to the gland surface to confirm the presence of trapped nitrogen-containing nutrient molecules before more biosynthetic resources are mobilized to produce the proteases and the slime molecules needed for its digestion in larger quantities. For example, when the traps are fed small, washed, non-digestible pebbles instead of digestible food particles, the traps close quickly and tightly, but begin to reopen after ~ 1.5 days instead of remaining closed (unpublished results). This suggests that the first, small batch of secreted proteases is used to test the trapped material for the presence of digestible food molecules. However, because a majority of the Golgi stacks are organized in clusters with their *trans* cisternae and TGN cisternae facing the cluster interior, getting the TGN cisternae with their budding secretory vesicles quickly out of the clusters and to the gland surface might require the involvement of an actin-based motility system. The translocation of Golgi stacks along actin filaments in plants was discovered ~ 20 years ago [30, 31] and has been studied extensively [32]. Less is known about the dynamics of free TGN cisternae, but in plants they are independently mobile cellular entities capable of delivering clusters of secretory vesicles to the cell surface [39, 40].

## Conclusion

During the past 10 years the combination of high pressure freezing and electron tomography techniques has transformed our understanding of the functional organization of the secretory apparatus of plant and algal cells [27, 33, 35]. In this study, we have extended this analysis to determine how the structural parameters of the ER, Golgi and TGN membrane systems in Venus flytrap gland cells change in response to the induction of lytic enzyme production and secretion. The principal finding of this investigation is that the secretory apparatus of resting (control) gland cells appears to be “overbuilt” to enable the gland cells to quickly up-regulate lytic enzyme secretion in response to the trapping of prey without having to first assemble new membrane systems. Most notably, the increase in synthesis of secretory proteases only requires the recruitment of more polysomes to ER membranes, and the existing ER-to-Golgi COPII vesicle transport system appears to possess excess transport capacity to accommodate the increased amounts of lytic enzymes produced in the ER. Similarly, the Golgi stacks are designed to accommodate the increased amount of slime production needed for delivering the enzymes in a hydrated medium to the trapped prey in the extracellular space. Finally, the discovery of loose bundles of actin-like filaments associated with free TGN cisternae exclusively in the 1 day post-induction samples suggests that actin-mediated secretory vesicle transport is used to speed up the initial vesicle transport to the cell surface and thereby the verification of the nutrient content of the trapped materials.

## Methods

### Stimulation of the gland cells

The glands of Venus flytrap, *Dionaea muscipula* (Sturtz and Copeland, Boulder, CO), were induced to secrete digestive enzymes by stimulation of the trigger hair cells with one large drop (100 µL) of 5% BSA solution that was placed into each trap.

### Collection of the flytrap extract for analysis

The secreted digestive enzyme extracts were obtained by gently removing the traps from their stems with a razor blade. Following removal of the midrib, the trap leaves were separated and the secreted materials (~ 70 µL per trap) recovered with a p200 pipetman and placed in an Eppendorf tube and stored at − 20 °C or freeze-dried.

### General protease assay

For the general protease assays a protease assay kit 539125 (Calbiochem, La Jolla, CA) was used. The assay utilizes a fluorescein thiocarbamoyl-casein derivative (FTC-casein) as a substrate. Protease activity cleaves FTC-casein into smaller peptides. The reaction is stopped with trichloroacetic acid (TCA), which causes the remaining FTC-casein to precipitate leaving the fluorescent peptides in the supernatant. The fluorescence was measured at 492 nm. The experiments were repeated three times with extracts from 20 traps for each day of the 6 days assayed.

### Zymogram activity gels

Zymograms were run as described previously [62, 63] with minor modifications. The 12% polyacrylamide SDS-PAGE gels contained 0.04% type I gelatin from porcine skin (Sigma, Saint Louis, MO) as substrate for proteases. Freeze-dried extracts from 20 flytraps per time point were rehydrated in distilled water up to the original volume. Once rehydrated, samples were mixed with the zymogram loading buffer [50 mM TRIS–HCl pH 6.8, 5 mM cysteine, 10% (v/v) glycerol, 0.2% (w/v) SDS, and 0.01% (w/v) bromophenol blue; 1 vol. sample: 2 vol. zymogram loading buffer]. The proteins were run with 5 µL sample per lane at 4 °C, at 23 mA for 2.5 h in a Mini Protean III gel system (Bio Rad, Hercules, CA). The proteins within the gels were renatured in the developing buffer (85 mM sodium acetate pH 4.0, 2.5% Triton X-100) for 30 min at 4 °C, and 30 min at room temperature. The gels were then incubated overnight (16–18 h) at 38 °C in developing buffer to enable the renatured proteases to digest the gelatin substrate (85 mM sodium acetate pH 4.0). A pH of 4.7 was also tried. Gels were stained in 0.05% Coomassie R-250 (50% water, 40% methanol, 10% acetic acid) and destained in a 10% acetic acid, 40% methanol solution.

Protease inhibitors (Sigma, Saint Louis, MO) were used to determine the specificity of the bands visualized by zymography. A zymogram was produced for each type of inhibitor. Samples were incubated with the inhibitor on ice for 20 min before loading. The inhibitor was also included in the washing and developing buffers. Cysteine proteases: l-*trans*-epoxysuccinyl-leucylamido (4-guanidino) butane (E-64) was prepared as indicated by supplier and 25 µM of the protease solution was included with the sample and in the washing and developing buffers. Aspartic proteases: Pepstatin was prepared as indicated by the supplier and 40 µM of the solution incubated with the sample and in the washing and developing buffers. Serine proteases: phenylmethylsulphonylfluoride (PMSF) was prepared as indicated by the supplier and 1 mM of the solution incubated with the sample, and 5 mM in the washing and developing buffers. Metallo proteases: phenanthroline was prepared as indicated by supplier and 10 mM of the solution incubated with the samples and buffers. A 5 mM solution of ethylenediaminetetraacetic (EDTA) was prepared with distilled water and used as an inhibitor. We also tested adding 5 mM EDTA to the sample buffers, and 10 mM to the gel and developing buffers.

### High-pressure freezing and freeze substitution

Individual traps from control and 1–6 day BSA-induced specimens were removed from the plant with a razor blade. Removal of the midrib of the leaf exposed the interior of each symmetric half of the trap. A disposable tissue punch (Technotrade, Manchester NH) was used to punch out a circle of gland tissue measuring approximately 1.9 mm in diameter. The bottom leaf cells on this circle were removed by sectioning at a diagonal to the gland cells. This thin disc of tissue was placed into an aluminum hat measuring 2 mm in diameter and 0.3 mm in height. 120 mM d-mannitol (Sigma, St. Louis, MO) or 130 mM sucrose (Mallinckrodt, Paris, Kentucky) was added to fill the volume of the hat. Freezing occurred in a BAL-TEC HPM-010 high-pressure freezer (Technotrade, Manchester, NH). Substitution was performed in 2% OsO_4_/0.1% uranyl acetate in anhydrous acetone (Ernest F. Fullam, Latham, NY) in cryovials (Nunc, Roskilde, Denmark) for 6 days in a metal block surrounded by dry ice at − 80 °C. The samples were slowly warmed to room temperature over the course of 2 days. During the course of this warming process, the samples slowly fell out of the hats. The room temperature samples were rinsed three times with anhydrous acetone and infiltrated with Epon resin (Ted Pella, Redding, CA) according to the following schedule: 1% resin in acetone (2 h), 2%, and 4% (2 h at each concentration), 8% resin, 16%, and 25% (12 h at each concentration), 50% resin, 75%, and 100% (24 h at each concentration). Polymerization was at 60 °C for 48 h in flat embedding molds.

### Sample preparation for electron microscopy and electron tomography

Thin sections were mounted on formvar-coated copper slot grids (EMS, Fort Washington, PA) and stained with 2% uranyl acetate in 70% methanol (10 min) and Reynold’s lead citrate (5 min). Approximately 200 EM micrographs of Golgi-containing cytoplasm were taken from control and 1–6 day BSA-induced glands, and analyzed in parallel with the tomograms. The 250 or 300 nm thick sections used for electron tomography were stained as above and mapped in a Philips CM10 (Phillips, Hillsboro, OR) microscope. The 15 nm colloidal gold (BBI International, Cardiff, United Kingdom) used as a fiducial marker was placed on either side of the grid for 15 min. After washing with distilled water, the grids were placed under a Technai F30 (FEI, Hillsboro, OR) microscope using 300 kV for acquisition of dual-axis ± 60° tilt series of the preselected cytoplasmic regions. Tomograms were produced of the control, 1-day, 4-day and 6-day samples. The images were aligned using the gold particles as fiducial markers in the manner described earlier [54]. An R-weighted back-projection algorithm was used to compute each tomogram [64]. Dual-axis tomograms from serial sections were joined to produce thicker tomograms and reconstructions. Of the ~ 160 cells examined, ~ 20 were subjected to tomographic analysis. Of these, 12 were chosen for slice-by-slice segmentation analysis yielding 32 dual-axis tomograms, which included 2950 ~ 2 nm slice images. Six cells from these were chosen for manual and detailed analysis using the IMOD program in a process called modeling. These reconstructions included 19 different Golgi stacks with a total of over 100 different Golgi cisternae, and a similar number of TGN elements. 3D modeling was performed as previously described [15, 65].

To illustrate the inherent variability in structure of the Golgi cisternae we have included a gallery of tomographic models of all cisternae studied (control, and 1-, 4- and 6-day BSA-induced) as Additional file 1: Figure S1.

## Additional file

**Additional file 1: Figure S1.** Gallery of tomographic reconstructions of Golgi stacks for each type of tomographic sample studied (control, and 1, 4 and 6 days BSA-induced). Each horizontal line represents a Golgi stack with *cis* cisternae colored orange and green, medial cisternae blue, pink and yellow, and *trans* cisternae colored purple and red. Within each group, the Golgi shown are from different cells. These data illustrate the inherent variability in structure of the Golgi cisternae. Bar 0.2 µm.
