# Supplementary figures and images for: 3D electron tomographic and biochemical analysis of ER, Golgi and trans Golgi network membrane systems in stimulated Venus flytrap (Dionaea muscipula) glandular cells
Source: J Biol Res (Thessalon). 2018 Aug 8;25:15. doi: 10.1186/s40709-018-0086-2 (PMC6083566; doi:10.1186/s40709-018-0086-2)

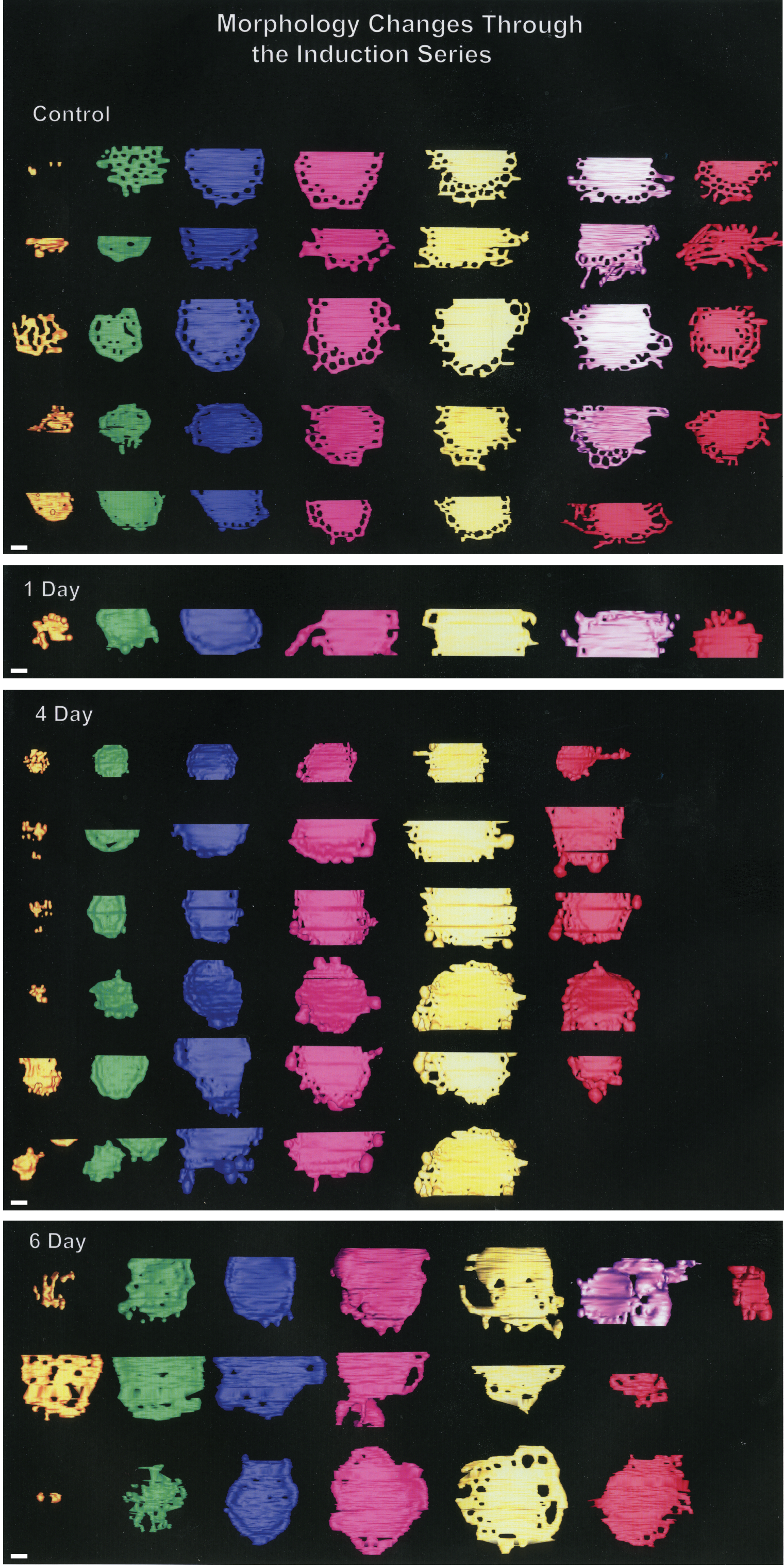

Supplement: Supplementary file 1 — Additional file 1: Figure S1. Gallery of tomographic reconstructions of Golgi stacks for each type of tomographic sample studied (control, and 1, 4 and 6 days BSA-induced). Each horizontal line represents a Golgi stack with cis cisternae colored orange and green, medial cisternae blue, pink and yellow, and trans cisternae colored purple and red. Within each group, the Golgi shown are from different cells. These data illustrate the inherent variability in structure of the Golgi cisternae. Bar 0.2 µm. [file 40709_2018_86_MOESM1_ESM.png]
